# Morbid Effects of Diseased Teeth upon the General System

**Published:** 1857-12

**Authors:** F. D. Thurman

**Affiliations:** Dentist, Atlanta, Ga.


### ARTICLE II.

*Morbid Effects of Diseased Teeth upon the General System.* By F. D. Thurman, Dentist, Atlanta, Ga.

As the teeth and their delicately constructed membranes are so directly connected by nerves and blood-vessels with the general system, it is not surprising that so many sympathetic affections should originate from this source, particularly when we take into consideration the fact, that the teeth are situated so near the brain, the locality of so many important nervous centers; and, also, the fact, that the periosteal tissue which surrounds the roots of the teeth (and is so often diseased) is but a reflection of the mucous membrane which lines the stomach, bowels, lungs, and whole internal viscera. But, notwithstanding the fact, that diseases of the head, throat, stomach, lungs, &c., so often originate with diseased teeth, it is only within the last few years that physicians are beginning to call the attention of the profession to the importance of the morbid relation existing between the teeth and other parts of the system; and even now, its importance is comparatively overlooked. They very often ascribe the cause of acute disease in children to the irritation produced by the eruption, or cutting of the temporary teeth; but whilst they seem well convinced that sound teeth pressing upon sound gum will produce acute diseases in children, they seem to overlook, in a measure, the important fact, that diseased teeth, with their inflamed membrane and vitiated secretions, may, upon a less irritable nervous system, produce chronic diseases of a most serious nature.

If we were not a degenerate race of people, and the constitution and health of mother and child were always perfect, we would never hear of such a thing as painful dentition; but, like the inferior animals, our teeth would frequently be ready for use before their presence had been observed. But, as it is otherwise, we must bear the penalty of violated laws of nature.

If the constitution and health of the child is perfect, nature is always ready and able to prepare the way for the growing tooth in a natural and painless manner. But children are seldom born with perfect constitutions, and those who approach nearest to it are generally rendered feeble by the inability of their delicate stomachs to digest the quality or quantity of milk it gets from an unhealthy or innocently imprudent mother, to say nothing of the *good things* it soon learns to cram, which gratify the palate and ruin the stomach.

A diseased or painful dentition generally shows itself by a swollen, inflamed and very sensitive condition of the gums, more or less fever and irritation of the bowels, flow of saliva, pressure of blood to the head, thirst, &c. These symptoms generally attend all of the milder forms of painful dentition. In the worst forms all of these symptoms are aggravated; the child starts and screams in its sleep, stupor and convulsions set in, and death often relieves the little sufferer.

*Treatment.*

Very frequently the child may be suddenly and effectually relieved by very carefully and perfectly separating the gums and membranes, and freeing the rising tooth. If the operation is not thoroughly performed it is worse than useless, as it only adds to the little patient's suffering without affording any relief. The state of the bowels should be very strictly attended to, and remedies administered according to the symptoms presented—such as mild purgatives, alkalies, cold and warm bathing, fresh air, &c., &c.

The eruption of the second set, or permanent teeth, is seldom attended with much pain or inconvenience, with the exception of the Dentes Sapientiæ, or wisdom teeth, which often produce a great deal of trouble, such as a high state of inflammation of the gums, which frequently extends to the throat and tracheo, and occasionally involving the lungs; fever, acute pain in the gums, headache, inflammation of the eyes, &c., &c.

In a large majority of cases relief may be obtained by freely separating the gums down to the tooth; but we sometimes find the tooth so crowded between the ramus or angle of the jaw and the second molar, that extraction is the only means of relief.

Diseases consequent upon decaying and diseased teeth are very numerous, though I shall not detail them, but simply mention a few general facts. Any one who has suffered much with the toothache, and felt its sudden and debilitating flashes throughout the nervous system, even to the extremity of the fingers, must feel convinced, (if he thinks at all,) that long continued diseases of the teeth and their membranes would produce like diseases of other parts, and in addition to their direct nervous sympathy we know that the food cannot be properly masticated upon diseased and partially decomposed teeth, and consequently it must pass into the stomach in a crude and unprepared state, taxing this organ with a double duty, a duty which nature never intended it to perform, and which will most assuredly tend to enfeeble the energies of the digestive powers, and as soon as the stomach loses its ability to digest the food properly, the whole man, both body and mind, becomes deranged and nervous, and other affections in various forms, real and imaginary, are the consequences.—The stomach is not only taxed with the double duty of mastication and digestion, but it is poisoned by the decomposed substance of the teeth, decayed animal and vegetable matter mixed with pus and vitiated secretions, which the food unavoidably collects in passing through the mouth. Now it is plain to be seen that we have a sufficient cause for dyspepsia in its worst form, and if we follow the consequences a little further, we see that the stomach, one of the main wheels in the human machine, is thrown out of gear: the whole machine must suffer. This undigested mass must produce more or less irritation of the whole intestinal canal. And now take a glance at the myriads of open mouths of the absorbent vessels, and observe, that, regardless of the propensity of this beautiful network system to choose between good and evil, it is forced (for want of a digestive power) to take up and conduct to the blood a portion of this extraneous compound.—The blood, in turn, is rendered impure and unfit for replenishing the decaying system with new and healthy flesh and bone.

Patent blood purifyers are now sought with avidity, which, if they were valuable at all, could only remove the effect for a time, whilst the cause is still operating. Eminent physicians are consulted, and with all of their profound learning and masterly skill, their penetrating glance seldom detects the impurities. They examine your cellars and other outhouses in search of decomposing substance and poisonous effluvium, whilst the large cavities in the molar teeth contain in the very worst form just what they are searching for. Now add to this the prostration of nervous influence consequent upon the severe pain connected with diseased teeth, and I think we have cause sufficient for many of the ills to which we are heir.—And I wish just here to notice again another very important consideration connected with the influence of diseased teeth upon the system, and that is the fact of this cause being so long continued. The hardest marble cannot withstand the continual dropping from the gentle brook. So with the human system. By its reacting and healing power it is able to withstand very severe shocks, which are short in duration, whilst causes which seem scarcely worthy of notice may, by their long continued action, bring about alarming results.

I might go on to describe many cases of sympathetic diseases of this kind that have come under my special notice, but as lengthy pieces are seldom read, I will close by simply advising physicians, particularly the young, to pay more attention to the state of their patients' mouths and teeth. When you look into your patient's mouth and detect with such unerring precision the slightest departure from a healthy standard in the mucous membrane of the tongue, don't forget that the teeth (notwithstanding they may be partially decomposed and surrounded by inflammation, suppuration and decaying substances,) are a part of the same organization.

